# Associations of Fecal Microplastics with Oxidative Damage and Cardiopulmonary Function: Evidence from a Pilot Study

**DOI:** 10.3390/toxics14010075

**Published:** 2026-01-14

**Authors:** Lili Xiao, Wenfeng Lu, Lan Qiu, Shuguang Wang, Jiayi Li, Jiayi Lai, Zhixuan Ji, Xiaoliang Li, Yun Zhou

**Affiliations:** 1 School of Public Health, Guangzhou Medical University, Guangzhou 511436, China; lilix@gzhmu.edu.cn (L.X.); karsawithl@163.com (W.L.); lane_q@126.com (L.Q.); wangshuguang1996@163.com (S.W.); ljy673006199@163.com (J.L.); ljy2541555818@163.com (J.L.); 13318366620@163.com (Z.J.); 2 Zhuhai Center for Chronic Disease Control and Prevention, Zhuhai 519060, China; lxlstudent@163.com

**Keywords:** fecal microplastics, oxidative damage, MDA, 8-OHdG

## Abstract

The ubiquity of microplastics (MPs) in the environment has raised significant concerns, yet their potential impacts on human health are not fully elucidated. This study aimed to quantify human exposure to MPs in feces and evaluate their associations with oxidative stress and cardiopulmonary function. A panel study was conducted in 16 male college students with three-round visits. Fecal MPs were quantified using infrared micro-spectroscopy, and health effects were assessed through urinary biomarkers of oxidative damage (MDA and 8-OHdG) and cardiopulmonary function tests. Associations between MP exposure and health outcomes were analyzed using linear mixed-effect models. We found that fecal MP amount across 48 samples from 16 participants showed high intra-individual variation and poor reproducibility (ICCs < 0.4). MPs in feces were predominantly identified as sheets and fragments in the 100–200 μm size range, with polyamide (PA), polyester, polyethylene (PE), and polypropylene as the primary polymer types. Significant relationships were observed between fecal MP amount and oxidative damage biomarkers. Each one-unit increase in MPs corresponded to a 0.827 increase in MDA (95% CI: 0.116, 1.54) and a 1.11 increase in 8-OHdG (95% CI: 0.235, 1.98), with fibrous shapes and specific polymers (PE and PA) being the primary drivers. No significant associations were found between MP exposure and lung function or blood pressure. These findings indicated that MP exposure was significantly linked to increased oxidative damage, highlighting a pressing public health concern regarding their subclinical biological effects.

## 1. Introduction

Microplastics (MPs) are ubiquitous environmental contaminants defined as plastic particles small than five millimeters [1]. They originate from diverse sources, including the fragmentation of larger plastic waste, the abrasion of tires and synthetic textiles, and the spillage of industrial pellets [1,2,3]. An additional source of MPs is household products, which can release particles from exfoliants in cosmetics and toothpaste, or from food packaging like water bottles and infant feeders [4,5]. The ubiquity of MP exposure has led to inevitable exposure in our daily life, raising significant public health concerns due to their potential adverse effects [4,6].

Recent evidence has suggested that MPs enter the human body through constantly ingesting and inhaling contaminated food, water, and air [3,7]. Once in contact with epithelial linings in the intestine or the lung, MPs may cumulatively cause physical, chemical, and microbiological toxicity [6,8,9]. However, whether MP exposure poses a substantial risk to human health remains poorly understood. A growing body of laboratory evidence from animal and cellular studies indicates that MPs can induce toxic effects [10,11,12]. For example, zebrafish exposed to MPs have exhibited growth retardation, oxidative damage, and gastrointestinal and metabolic disturbances [13]. Similarly, in vitro studies have also reported that MP exposure causes a range of detrimental effects, from immune response and cytokine secretion to oxidative stress, cellular stress, and programmed cell death [11,14]. In light of the limited direct evidence regarding the human health effects of MPs, epidemiological research on MP exposure is now an urgent international priority.

The compositions of MPs vary widely in shape, size, and polymer type, reflecting their diverse sources and production processes [15,16]. Most laboratory studies only exposed cells or animals to one type of MPs, such as polystyrene (PS) spheres, which actually cannot represent the diversity and amount that humans expose [11,12,14]. In addition, because of the different routes and durations of exposure, internal levels of MPs may vary dramatically among individuals [17,18]. Previous studies have established that MPs are primarily eliminated via feces after passing through the gastrointestinal tract [19,20,21]. The presence and abundance of MPs in feces serves as a relatively accurate and non-invasive biomarker for assessing human exposure [22].

In this study, we conducted a pilot study to assess potential adverse health effects of MP exposure. Specifically, we measured fecal MP amount as well as its properties, including various shapes, sizes, and chemical types. As predominant forms of oxidative lesions, urinary malondialdehyde (MDA) and 8-hydroxy-2′-deoxyguanosine (8-OHdG) are critical biomarkers for oxidative DNA damage and lipid peroxidation. We further investigated associations between fecal MP levels and oxidative stress biomarkers (MDA and 8-OHdG), lung function, and blood pressure to assess the adverse health effects of fecal MP exposure.

## 2. Materials and Methods

### 2.1. Study Participants

**Figure 1:**
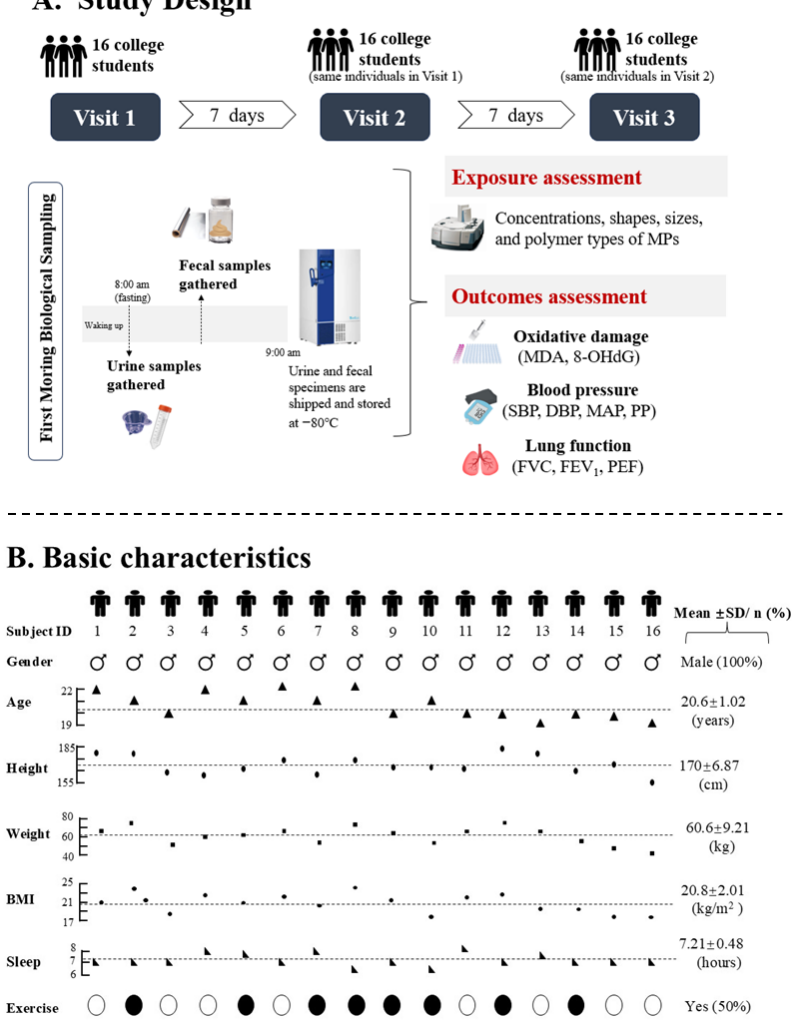
Study design and participant characteristics. (**A**) Schematic overview of the panel study design, illustrating timeline of fecal microplastic exposure assessment and measurements of oxidative damage and cardiopulmonary function. (**B**) Bar graph summarizing the baseline demographic characteristics of study participants.

A pilot study of college students from Guangzhou Medical University (Guangzhou, China) was conducted during three consecutive weeks in November 2020 (shown in Figure 1A). Volunteer students were excluded if they had a habit of cigarette smoking or alcohol drinking; they suffered from any clinically diagnosed chronic disease including respiratory disease (International Classification of Diseases 10th Revision (ICD-10): J00-J99), cardiovascular disease (ICD-10: I00-I99), and infectious and parasitic diseases (ICD-10: A00-B99); they had any medication use in the past four weeks. A total of 16 male college students living in the same dormitory building were finally included in this study. All the participants stayed in the campus during the study period. Data on demographics and anthropometrics of each participant were collected. Physical examinations on cardiopulmonary indicators (blood pressure and lung function) and biological samples including morning urine and feces were collected at each visit. The research protocol was approved by the Ethics Committee of Guangzhou Medical University (202008030), and all participants provided written informed consent before participation.

### 2.2. Assessment of Fecal MP Exposure

In the study, feces samples were collected following the participants’ first defecation time. Each participant was asked to put on medical-grade gloves and lay the collection area out with a new aluminum foil before defecation. Feces were directly defecated on the aluminum foil and participants were instructed to gently take five scoops of feces into a wide-neck glass container which was subsequently hermetically sealed with wooden lids. The extraction and detection of fecal MPs were performed on a cleanroom bench according to previous studies [19,23]. Briefly, feces samples were dried with a vacuum freeze-dryer to remove the moisture content of feces. Then the lyophilized fecal samples were weighed and dissolved with 100 mL of 30% (*v*/*v*) H_2_O_2_ on a reciprocating shaker for two weeks to remove most non-plastic matter such as high amounts of solids, bacterial biomass, proteins, fat, and mucus. After dissolution, saturated salt solution (1.6 kg/L zinc chloride) was added at a volume ratio of 1:2 for density floatation of MPs. The obtained solution was filtered through vacuum filtration with glass membrane filters (47 mm diameter and 1.0 μm pore size). Filters that collected suspected particles were rinsed with ultrapure Milli-Q water three times and subsequently air-dried at 50 °C for 24 h in a clean Petri dish.

The abundance and properties of MPs were identified through micro-Fourier transform infrared (micro-FTIR) spectroscopy (Nicolet™ iN10, Thermo Fisher Scientific, Waltham, MA, USA). Target particles were scanned to collect infrared characteristic spectra, which were recorded in the spectral range of 650–4000 cm^−1^. The vibrations of the functional groups in target particle spectra were observed as to their homologous infrared absorption peaks with an in-house library, and 70% spectral similarity was adopted as a threshold to identify the characteristics of infrared spectra. Fecal MP levels were quantified as the number of confirmed MP particles per gram of dry fecal mass (pieces/g dm). Properties of MPs including shape, size and chemical types were also observed and recorded.

For quality-control purposes, participants were trained to perform fecal collection with the above uniform procedures. All chemical solutions were filtered through a 50 μm metal sieve, and laboratory equipment was prerinsed with ultrapure Milli-Q water before use to minimize contamination by foreign particles. Ultrapure Milli-Q water samples were also treated as the quality control and measured along with feces samples through all the steps of the procedure to test for potential contamination; they ultimately tested negative for the presence of MPs.

### 2.3. Health Measurements

Oxidative damage biomarkers: Morning urine samples were stored at −20 °C in a freezer until analysis. To ensure the stability of oxidative damage biomarkers, urinary specimens were measured within six months of collection. Thawed urine samples were centrifuged at 3000× *g* for 10 min and supernatants were used in the further processing. The concentrations of oxidative markers, urinary 8-OHdG and MDA, were detected using commercially available enzyme-linked immunosorbent assay (ELISA) kits (KA5026, Abnova, Tianwan, China; AD11335Hu, Andy Gene, Beijing, China). Both tests of 8-OHdG and MDA were carried out in triplicate according to the manufacturer’s protocols. The detection range of ELISAs for urinary MDA and 8-OHdG were 0.2 to 7.5 pg/mL and 62.5 to 8000 pg/mL, respectively. Intra-assay coefficients of variance for urinary MDA and 8-OHdG were less than 10%. Both urinary MDA and 8-OHdG concentrations were standardized by urinary creatinine (Cr) levels to correct for differences in urine dilution. In the current study, Cr-corrected urinary MDA and 8-OHdG were expressed as nmol/mg Cr and ng/g Cr, respectively.

Lung function: Trained technicians measured the lung function of participants using an electronic spirometer (Chestgraph HI-101, Chest M.I. Inc., Tokyo, Japan) in accordance with the American Thoracic Society recommendations [24]. Each participant remained in a sitting position and was requested to perform the test following oral instructions from technicians. A minimum of three acceptable trials from a maximum of eight maneuvers were obtained. Measurements including forced vital capacity (FVC, L), forced expiratory volume in 1 s (FEV_1_, L), and peak expiratory flow (PEF, L/s) were used as lung function indicators.

Blood pressure: Participants were instructed to sit in a room for at least five minutes and then had their left upper arm blood pressure measured using an electronic sphygmomanometer (Omron, Tokyo, Japan). Systolic blood pressure (SBP) and diastolic blood pressure (DBP) were obtained based on readings of the machine. Each participant was measured three times with 2 min intervals between each measurement. SBP and DBP were recorded on the average of three sets of readings. Pulse pressure (PP) and mean arterial pressure (MAP) were calculated using formulas of the SBP and DBP as [SBP − DBP] and [MAP = (DBP × 2 + SBP) ÷ 3], respectively.

### 2.4. Statistical Analysis

Basic characteristics of the participants were described as proportions for categorical data and mean ± standard deviation (SD) for normally distributed data. We acquired descriptive statistics to characterize the distribution of MP levels in feces and plotted a chart to visually compare mean levels of fecal MPs between participants. Intraclass correlation coefficients (ICCs) were calculated to assess the reproducibility of fecal MP levels across the three sampling rounds. The analysis included 48 samples from 16 male college students collected over an approximately one-month period. Pie graph and percentage bar graph were plotted to demonstrate the overall and between-person frequency distributions for the shape, size, and polymer types of fecal MPs, respectively. Median (interquartile range) and mean ± SD were used to assess the descriptive distributions of oxidative damage and cardiopulmonary endpoints, respectively. We constructed a dichotomous variable by classifying levels of fecal MPs as low and high MP exposure groups with the median value as the cut-off point. Differences in oxidative damage and cardiopulmonary endpoints between low- and high-exposure MP groups were estimated by Student’s *t*-test and the Mann–Whitney U test according to the data distribution.

We constructed linear mixed models to examine the association of MP exposure with oxidative damage and cardiopulmonary endpoints. This method incorporated random intercepts for each participant to account for intra-individual correlations between repeated measurements, adjusting within-person variance that changes over time. The linear mixed model also included fixed-effect covariates: age (continuous), BMI (continuous), sleep duration (continuous), and physical activity (yes/no). Associations were quantified by calculating regression coefficients (β) and 95% confidence intervals (CI), where fecal MP levels were analyzed as a continuous or dichotomous variable. Due to right-skewed distributions, urinary MDA and 8-OHdG levels were log-transformed before modeling. To investigate the associations between properties of MP exposure and oxidative damage and cardiopulmonary endpoints, we coded various shapes, size, and polymer types of MPs as dummy variables and estimated their β and 95% CI in the model. False Discovery Rate (FDR) correction was applied to adjust for multiple comparisons. All data analyses were performed using R version 4.2.0 and 2-sided *p* < 0.05 was considered as statistically significant.

## 3. Results

### 3.1. Basic Characteristics of Study Participants

Study participants were 16 males and their basic characteristics are presented in Figure 1B. The mean age of the 16 participants was 20.6 (SD: 1.02) years and the mean BMI was 20.4 (SD:2.63) kg/m^2^. Half of the participants partake in regular physical activity and the mean value of their sleep duration was 7.14 (SD: 0.49) hours. All 16 participants completed three-round visits and 48 feces samples were obtained.

### 3.2. Distributions of MP Exposure in Feces

Table 1 presents the fecal MP amounts from 16 participants across three sampling rounds. The distributions varied, with median values of 35, 30, and 36.5 pieces/g dry mass for rounds 1, 2, and 3, respectively. In total, the fecal MP amount across the 48 samples ranged from 8 to 88 pieces per gram dry mass (pieces/g dm). The median value of fecal MPs in the 48 samples was 34.5 pieces/g dm, with a mean level of 40.1 pieces/g dm. The average amount of fecal MPs for each participant is presented in Figure S2, and half of the participants had higher fecal MP levels than the average.

Table 2 shows the variability of fecal MP amount over three-round visits. Serial measurements of fecal MP amount on three-round visits showed poor correlations and reproducibility. The Spearman correlation coefficients were −0.227 for the one-week period and 0.096 for the two-week period, which did not reach statistical significance (both *p* values > 0.05). Additionally, poor reproducibility was observed for serial measurements of fecal MP amount over the three-round visit, with ICC values of −0.144 (95% CI: −0.611, 0.374) for the one-week period, and 0.0136 (95% CI: −0.513, 0.506) for the two-week period.

**Figure 2:**
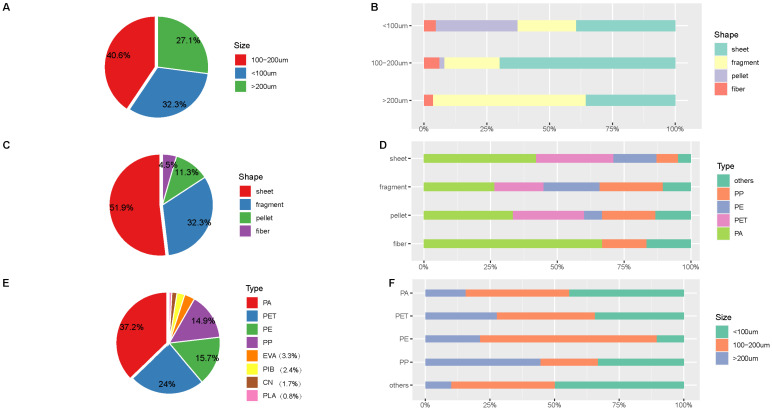
The overall frequency distributions for the shapes (**A**), size (**C**), and polymer types (**E**) of fecal MPs, and shape distribution of each size detected (**B**), polymer type distribution of each shape (**D**), size distribution (**F**) of each polymer type in feces. Abbreviations: PA, polyamide; PET, polyester; PE, polyethylene, PP, polypropylene. Those with relative proportions less than 10% were classified as others, including ethylene vinyl acetate copolymer (EVA), polyisobutylene (PIB), cellulose nitrate (CN), and polylactic acid (PLA).

The size of the MPs ranged from 15 to 500 μm. MPs detected in the feces were dominated by the range between 100 and 200 μm, with a relative proportion of 40.8% (shown in Figure 2A). Four MP shapes, including sheets, fragments, pellets, and fibers, were identified in all feces samples (shown in Figure S1). The identified MPs were mostly shaped as sheets and fragments, with proportions of 51.9% and 32.3%, respectively (Figure 2C). Four main chemical types of MPs were identified, polyamide (PA), polyester (PET), polyethylene (PE), and polypropylene (PP), with relative proportions of 34%, 22%, 16%, and 14%, respectively (Figure 2E). Additionally, different sizes of MPs were dominated by different shapes and polymer types (Figure 2B,D,F). For example, MPs of 100–200 μm were dominated by sheets, whereas MPs more than 200 μm were dominated by fragments. Similar distributions of shape, size, and chemical types were observed for each participant (Figure S3) and each visit (Table S1).

### 3.3. Associations Between Fecal MP Amount and Health Endpoints

Because a single fecal MP measurement provides only a snapshot of exposure, we merged data from all three sampling rounds for analysis. This resulted in a combined dataset of 48 observations with repeated measures from 16 participants. Among the 48 observations, median values of the oxidative damage biomarkers urinary MDA and 8-OHdG were 2.26 nmol/mg Cr and 99.8 ng/g Cr, respectively (shown in Table S2). There was an appreciable increase in the levels of urinary MDA and 8-OHdG in the high-MP-exposure group (2.94 nmol/mg Cr and 103 ng/g Cr) compared to those (1.85 nmol/mg Cr and 57.8 ng/g Cr) of the low-MP-exposure group (*p* < 0.05) (shown in Table 3). However, no statistically significant differences were observed in the lung function and blood pressure indicators between the high- and low-MP-exposure groups.

In the mixed-effect model analysis, each 1-piece/g dm increase in fecal MP level was significantly associated with a 0.827 (95% CI: 0.116 to 1.54) and 1.11 (95% CI: 0.235 to 1.98) increase in MDA and 8-OHdG (shown in Table 4). The categorical analysis also showed significant positive associations between fecal MP levels and both MDA and 8-OHdG (all *p* < 0.05). No significant associations were observed for lung function and blood pressure indicators with fecal MP levels.

Associations of oxidative damage, lung function, and blood pressure indicators with shapes, sizes, and the main four chemical types of MPs are presented in Tables S3–S5. Positive associations were observed between shapes, sizes, and polymer types of MPs and oxidative damage biomarkers, although not all were statistically significant. Prior to multiple comparison correction, several MP characteristics showed nominal associations with oxidative damage; significant effects on MDA were associated with fiber-shaped MPs and PE and PA types (*p* < 0.05). For 8-OHdG, significant effects were observed to be associated with MPs of size 150 μm to 200 μm and PE type (*p* < 0.05, shown Table S3). After applying FDR correction, none of the associations remained statistically significant. No significant associations were observed between fecal MP characteristics (shape, size, chemical type) and lung function or blood pressure, regardless of whether *p*-values were corrected or uncorrected for multiple comparisons (shown in Tables S4 and S5).

## 4. Discussion

In the present study, we found that MP exposure in feces was predominantly identified as sheets and fragments in the 100–200 μm size range, with PA, PET, PE, and PP as the primary polymer types. Fecal MP measurements showed poor longitudinal reliability for individuals, as indicated by ICC estimates of less than 0.2. A single measurement of fecal MPs offers merely a snapshot of an individual’s exposure level at the time of collection. Higher fecal MP levels were significantly associated with increased MDA and 8-OHdG, suggesting that MP exposure may contribute to oxidative damage relevant to human health.

Human exposure to MPs along with resultant potential health effects has attracted worldwide attention, which is a major environmental concern on the public. However, few published studies have yet directly assessed the human risk of MPs that need to be addressed to move forward. Therefore, our study exploring the health effects of MPs has been of great importance with substantial implications for public health. It has been reported that risk assessment of MPs on humans can be drawn from a parallel of particulate matter, which triggers oxidative stress and leads to increased risk of cardiovascular and respiratory diseases [17,25]. Our findings highlight the public health concern regarding the early mechanic biological endpoints, i.e., oxidative damage, but also apical endpoints from lung function and blood pressure. Effective strategies are urgently needed to control the lifetime inevitable exposure to MPs, preventing the public from the potential hazards of MPs to human health.

Previous experimental studies have reported the potential of MPs to cause a variety of biological effects, among which oxidative stress is of high importance as a critical response underlying the toxicity of MPs [26]. Excessive reactive oxygen species (ROS) production was observed in marine organisms after MP exposure, resulting disruption of redox homeostasis and increasing levels of oxidative damage biomarkers such as malondialdehyde, protein carbonyls, and lipid peroxidation [27,28]. An assessment of the toxicity of PP in human derived cells (HDFs, PBMCs, Raw 264.7) suggested that higher MP exposure amounts can simulate oxidative damage via an increase in the level of ROS [29]. However, possibly due to limited information on human exposure of MPs, no epidemiological studies have yet investigated oxidative damage effects of MP exposure for humans. In the present study, we detected MP burden in human feces, which was relatively higher than the amount of fecal MPs (9.4 pieces/g) in eight adults from Asia and Europe [19]. The main reason for the apparent discrepancy between our study and these two studies might be the moisture content of feces, which greatly affected the amount of MP burden in human feces [30,31]. We detected fecal MP amount based on the dry mass of feces, but fecal MPs in the previous study were detected using the wet weight of feces. Agreeing with our results, Yan et al. detected the MP burden in a dry mass of human feces and reported an average amount of fecal MPs reaching 34.9 pieces/g dm [20]. However, they did not further explore the associations between fecal MP burden and biological effects. Our results showed significant associations between higher fecal MP amount and increased MDA and 8-OHdG, firstly providing epidemiological evidence that high fecal MP burden is associated with oxidative damage.

In the present study, we quantitatively and qualitatively assessed fecal microplastics, including their amount, shape, size, and polymer type. Previous laboratory studies exposed cells or animals to MPs using commercially available particle types such as spherical PS or PE with up to two or three sizes, which might fail to capture the actual exposure of MPs with diversity in shapes, sizes, and polymer types [32,33,34]. Our study reported five shapes and four main polymer types of fecal MPs with sizes between 15 and 500 μm in male college students. To date, only three published studies have presented highly diverse shapes, sizes, and polymer types of MP exposure in human feces, which were partly consistent with our results [19,20,21]. For example, PA and PET MPs were the main polymer types detected in feces between our study and previous studies.

Potential hazards that MPs pose to organisms might be dependent on their unique chemical polymer types. In the present study, we found that PA and PE were significantly associated with MDA and 8-OHdG amounts. PE has been found as the main kind of MP polymer type and is frequently used as the representative MP in experimental studies to observe related effects [35,36]. Consistent with our findings, Zhang et al. conducted a study in crayfish with dietary exposure to PE particles and found that PE accumulated in the intestine of crayfish and caused oxidative stress responses, indicated by increased levels of oxidative-stress-related biomarkers [37]. However, related toxicological effects of PA have rarely been assessed, compared with other types of MPs such as PP and PE, though PA is also one of most abundant MPs in drinking water, marine animals and the human gut [38,39,40]. PA is an important thermoplastic with amide linkage in the polymer backbone and is widely used in fiber materials and engineering plastics [41,42]. Our study revealed a significant association between PA exposure and MDA, indicating that PA may contribute to the oxidative damage associated with MP exposure.

Apart from polymer types of MPs, physical properties of MPs were also found to be associated with oxidative damage in the present study. Our results indicated that fiber-shaped MPs contributed to increased levels of oxidative damage biomarkers. Exposure to fiber-shaped MPs is mainly derived from laundering synthetic textiles and tire erosion particles in our daily life [43]. Previous studies reported that adverse health effects of fiber-shaped MPs were observed in aquatic organisms, including tissue damage, reduced growth, and negative effects on body condition and even mortality [44]. Agreeing with our findings, higher MDA levels were observed in workers of a fiber textile factory than in controls without any exposure [45]. In addition, MPs with different size distributions were also detected in the present study. Similarly to our study, accumulating evidence suggested that MPs of size between 15 and 500 μm were detected in the feces of mussels exposed to MPs [46]. Previous studies indicated that the size distribution of MPs was an important factor in the toxicity of MPs, where size-dependent adverse effects of MPs were observed in adult marine medaka exposed to different sizes (2, 10, and 200 μm) of PS [47]. However, they did not assess related effects of MPs with size larger than 200 μm. In the present study, we comprehensively explored the associations between MPs ranging from 15 to 500 μm and related health effects, and found that MPs with size between 100 and 200 μm were significantly associated with oxidative damage in the present study. Noteworthily, no significant associations of MP exposure with lung function and blood pressure were found. It has been reported that microplastic particles less than 10 μm have been found to be translocated to other tissues or organs via the transportation of the circulatory system [48,49]. Due to the limitation of our analysis method, we did not detect a burden of fecal MPs less than 15 μm in the present study. Further studies with advanced detection methodology are needed to explore cardiopulmonary effects of MPs with size less than 15 μm.

There were several strengths to our study. First, this study provided epidemiological evidence for the associations between MP exposure and oxidative damage and cardiopulmonary indicators, moving a step forward to fill the data gap on the human risk assessment of MPs. Additionally, not only fecal MP amount but also their corresponding shapes, sizes, and chemical polymer types were quantitatively and qualitatively detected, comprehensively assessing potential adverse health effects of MP exposure. Finally, continuous and categorical statistical models were constructed to verify the robustness of our findings, and bias of results was reduced by strict quality control in data collection and experimental measurement. However, some limitations needed to be acknowledged in the present study. The main limitation was the limited sample size of the panel study, which might refrain the ability of our study to obtain more powerful results; thus, caution should be exercised in interpreting our results. Second, our study only included the young male population, limiting the generalizability of our findings. More studies with different ages and females are needed in the future to analyze the health effects of MPs. Finally, data on external exposure of MPs were not investigated; further studies on different sources of MP exposure are warranted for more accurate health risk assessment of MPs.

## 5. Conclusions

Our study provides evidence of a significant association between fecal MP exposure and increased oxidative damage, revealing a measurable subclinical effect linked to MP exposure in humans. Further investigation with larger samples will be required to confirm our findings and explore their potential effects on diseases.

## Figures and Tables

**Table 1:** Distributions of fecal MP amounts for 16 participants during three-round visits.

Periods	Samples	Mean	GM	Percentiles of Fecal MP Amounts (pieces/g dm)				
				Min	P_25_	P_50_	P_75_	Max
Visit1	16	41.1	36.3	17	22.0	35.0	58.8	71
Visit 2	16	35.2	29.8	8	20.2	30.0	53.0	84
Visit 3	16	44.0	38.7	13	28.2	36.5	60.2	88
Total	48	40.1	34.7	8	22.0	34.5	57.3	88

Abbreviations: GM, geometric mean; MP, MPs. The unit of fecal MP amounts was pieces/g dm, representing the number of confirmed MPs per dry mass of feces.

**Table 2:** Variability of fecal MP amounts across study periods.

	r/ICC (95% CI)	*p* Value
Spearman correlation coefficients		
One-week period	−0.227	0.399
Two-week period	0.096	0.724
Intra-class correlation coefficients		
One-week period	−0.144 (−0.611, 0.374)	0.704
Two-week period	0.0136 (−0.513, 0.506)	0.480

Abbreviations: r, Spearman correlation coefficient; ICC, intra-class correlation coefficient; CI, confidence interval; MP, microplastic.

**Table 3:** Differences in oxidative damage and cardiopulmonary endpoints between low and high fecal MP groups (N = 48).

Health Endpoints	Low Group (MN < 34.5 pieces/g dm)	High Group (MN ≥ 34.5 pieces/g dm)	*p*
Oxidative stress			
MDA (nmol/mg Cr, median (IQR))	1.85 (1.08)	2.94 (3.12)	0.029
8-OHdG (ng/g Cr, median (IQR))	57.8 (83.1)	103 (102)	0.012
Lung function			
FVC (L, mean ± SD)	4.02 ± 0.52	3.75 ± 0.74	0.155
FEV_1_ (L, mean ± SD)	3.56 ± 0.44	3.33 ± 0.64	0.150
PEF (L/s, mean ± SD)	7.49 ± 1.24	6.76 ± 1.96	0.133
Blood pressure			
SBP (mmHg, mean ± SD)	115 ± 11.8	112 ± 8.13	0.313
DBP (mmHg, mean ± SD)	71.9 ± 8.34	70.0 ± 6.67	0.386
PP (mmHg, mean ± SD)	43.1 ± 7.04	40.9 ± 6.24	0.250
MAP (mmHg, mean ± SD)	86.3 ± 9.04	84.5 ± 6.17	0.421

Abbreviations: MDA, malondialdehyde; 8-OHdG, 8-hydroxy-2′-deoxyguanosine; FVC, forced vital capacity; FEV_1_, forced expiratory volume in 1 s; PEF, peak expiratory flow; SBP, systolic blood pressure; DBP, diastolic blood pressure; PP, pulse pressure; MAP, mean arterial pressure; IQR, interquartile range. Data are presented as median (interquartile range) for non-parametrically distributed data, or mean ± SD for parametrically distributed data. Difference in oxidative damage and cardiopulmonary endpoints between low and high groups were estimated by Student’s *t*-test and Mann–Whitney U test according to the data distribution.

**Table 4:** Associations of fecal MP amount with oxidative damage and cardiopulmonary function.

Health Endpoints	Regression Coefficients (95% CI) by Dichotomy of Fecal MP Amount		Regression Coefficients (95% CI) by Continuous Fecal MP Amount (pieces/g dm)	*p*
	Low Group (MN < 34.5 pieces/g dm)	High Group (MN ≥ 34.5 pieces/g dm)		
Oxidative stress				
MDA	0 (reference)	3.46 (0.515, 6.49)	0.827 (0.116, 1.54)	0.028
8-OHdG	0 (reference)	4.82 (0.928, 8.87)	1.11 (0.235, 1.98)	0.042
Lung function				
FVC	0 (reference)	−0.010 (−0.911, 0.891)	−0.003 (−0.119, 0.113)	0.521
FEV_1_	0 (reference)	−0.057 (−0.201, 0.079)	−0.009 (−0.082, 0.064)	0.640
PEF	0 (reference)	−0.039 (−0.135, 0.057)	−0.007 (−0.046, 0.032)	0.597
Blood pressure				
SBP	0 (reference)	−0.338 (−0.851, 0.175)	−0.023 (−0.111, 0.065)	0.698
DBP	0 (reference)	−0.152 (−3.77, 3.07)	−0.015 (−0.059, 0.029)	0.751
PP	0 (reference)	−0.284 (−0.639, 0.071)	−0.019 (−0.065, 0.027)	0.794
MAP	0 (reference)	−0.120 (−0.416, 0.176)	−0.010 (−0.038, 0.018)	0.762

Abbreviations: MDA, malondialdehyde; 8-OHdG, 8-hydroxy-2′-deoxyguanosine; FVC, forced vital capacity; FEV_1_, forced expiratory volume in 1 s; PEF, peak expiratory flow; SBP, systolic blood pressure; DBP, diastolic blood pressure; PP, pulse pressure; MAP, mean arterial pressure. Fecal MP amounts were classified as low- and high-MP-exposure groups at the reference of median values. Linear mixed-effect models were constructed to examine the associations of MP exposure with oxidative damage and cardiopulmonary endpoints in both continuous and categorical models, with adjustment for age (continuous), BMI (continuous), sleep duration (continuous), and physical activity (yes/no) as fixed-effect covariates. In our model, a one-unit increase in the exposure variable corresponds to an increase in one MP particle per gram of dry fecal mass (1 piece/g dm).

## Data Availability

The original contributions presented in this study are included in the article/Supplementary Materials. Further inquiries can be directed to the corresponding author.

## Supplementary Materials

The following supporting information can be downloaded at: https://www.mdpi.com/article/10.3390/toxics14010075/s1, Table S1. Distributions of fecal MP shapes, sizes, and polymer types for 16 participants during three-round visits. Table S2. Descriptive distributions of oxidative damage and cardiopulmonary function among 16 male college students with three-round visits. Table S3. Associations of the shape, size, and chemical types of fecal MPs with oxidative damage. Table S4. Associations of the shape, size, and chemical types of fecal MPs with lung function. Table S5. Associations of the shape, size and chemical types of fecal MPs with blood pressure. Figure S1. Four shapes of microplastics detected in feces. Figure S2. Average concentrations of microplastics in feces by 16 young male college students with three visits (N = 48). The line chart was plotted to characterize distributions of fecal microplastics levels between persons. Bars represented mean level for each subject labeled 01 to 16. The red dotted line was the overall mean concentration of 48 samples with a value being 40.1 pieces/g dm. Figure S3. Distributions for the shape, size, and chemical type of fecal microplastics in 16 male college students with three-round visits. Percentage bar graph depicted between-person frequency distributions for the shape, size, and chemical types of fecal microplastics. PA, polyamide; PET, polyester; PE, polyethylene, PP, polypropylene. Those with relative proportions less than 10% were classified as others, including polyisobutylene (PIB), polylactic acid (PLA), cellulose nitrate (CN), ethylene vinyl acetate copolymer (EAA).
