# Supplementary material for: Associations of Fecal Microplastics with Oxidative Damage and Cardiopulmonary Function: Evidence from a Pilot Study
Source: Toxics. 2026 Jan 14;14(1):75. doi: 10.3390/toxics14010075 (PMC12846062; doi:10.3390/toxics14010075)
Supplement: Supplementary file 1 [file toxics-14-00075-s001.zip › toxics-4082137-supplementary.pdf]

Supplement

Associations of fecal microplastics with oxidative damage and cardiopulmonary function: evidence from a pilot study

Lili Xiao [1,†], Wenfeng Lu [1,†], Lan Qiu[1], Shuguang Wang [1], Jiayi Li [1], Jiayi Lai [1], Zhixuan Ji [1], Xiaoliang Li [2], Yun Zhou [1,*]

Affiliations

[1] School of Public Health, Guangzhou Medical University, Guangzhou, Guangdong 511436, China; lilix@gzhmu.edu.cn (L.X.); karsawithl@163.com (W.L.); lane_q@126.com (L.Q.); wangshuguang1996@163.com (S.W.); ljy673006199@163.com (Jiayi Li); ljy2541555818@163.com (Jiayi Lai); 13318366620@163.com (Z.J.)

[2] Zhuhai Center for Chronic Disease Control and Prevention, Zhuhai, Guangdong 519060, China; lxlstu-dent@163.com (X.L.)

*Correspondence: yunz@gzhmu.edu.cn (Y.Z.); Tel.: +86 13828456209 (Y.Z.).

†These authors contribute equally to this work.

Table S1. Distributions of fecal MP shapes, sizes, and polymer types for 16 participants during three-round visits

Characteristics of MPs	Total	Visit 1	Visit 2	Visit 3	*P*
**Shapes**					
sheet	69(51.9%)	18(39.1%)	27(65.8%)	24(52.2%)	0.273
fragment	43(32.3%)	19(41.3%)	9(22.0%)	15(32.6%)	
pellet	15(11.3%)	6(13.1%)	3(7.3%)	6(13.0%)	
fiber	6(4.5%)	3(6.5%)	2(4.9%)	1(2.2%)	
**Sizes**					
<100μm	43(32.3)	15(32.6%)	13(31.7%)	15(32.6%)	0.964
100-200μm	54(40.6%)	19(31.7%)	18(43.9%)	17(37.0%)	
≥200μm	36(27.1)	12(43.9%)	1024.4%)	14(30.4%)	
**Main polymer types**					
PA	45(37.2%)	15(35.7%)	14(35.9%)	16(40.0%)	0.921
PET	29(23.9%)	10(23.8%)	11(28.2%)	8(20.0%)	
PE	19(15.7%)	5(11.9%)	7(17.9%)	7(17.5%)	
PP	18(14.9%)	7(16.7%)	4(10.3)	7(17.5%)	

Abbreviations: PA, polyamide; PET, polyester; PE, polyethylene, PP, polypropylene.

Data are presented as n (%).

Table S2. Descriptive distributions of oxidative damage and cardiopulmonary function among 16 male college students with three-round visits

Endpoints	Percentiles of oxidative damage and cardiopulmonary endpoints						
	Min	P_5	P_{25}	P_{50}	P_{75}	P_{95}	Max
**Oxidative damage**							
MDA, nmol/mg Cr	0.56	0.87	1.37	2.26	3.93	9.26	11.8
8-OHdG, ng/g Cr	4.01	7.68	46.55	99.79	132.96	324.92	359.86
**Lung function**							
FVC, L	2.23	2.65	3.57	3.94	4.19	5.03	5.46
FEV_1, L	1.63	2.46	3.22	3.43	3.76	4.3	4.57
PEF, L/s	2.37	3.59	6.01	7.47	8.06	8.98	9.16
**Blood pressure**							
SBP, mmHg	85	95.7	104.75	113	117.75	127.65	132
DBP, mmHg	54	60.35	66.75	69	75	84.3	87
PP, mmHg	28	30	36	40.5	46	51.65	55
MAP, mmHg	64.33	72.68	79.83	84	89	98.1	102

Abbreviations: MDA, malondialdehyde; 8-OHdG, 8-hydroxy-2′-deoxyguanosine; FVC, forced vital capacity; FEV_1, forced expiratory volume in 1 s; PEF, peak expiratory flow; SBP, systolic blood pressure; DBP, diastolic blood pressure; PP, pulse pressure; MAP, mean arterial pressure.

Table S3. Associations of the shape, size, and chemical types of fecal MPs with oxidative damage

Variables	Percent change (95% CI) in MDA[#]	*P*	FDR	Percent change (95% CI) in 8-OHdG[#]	*P*	*FDR*
**Shapes**						
sheet	1.91 (-5.27, 9.63)	0.789	0.789	4.61 (-5.19, 15.5)	0.796	0.796
fragment	2.16 (-2.87, 7.45)	0.517	0.689	1.64 (-4.96, 8.71)	0.458	0.796
pellet	1.85 (-2.21, 6.07)	0.414	0.689	1.73 (-6.12, 10.2)	0.611	0.796
fiber	4.00 (2.24, 5.78)	**0.038**	0.152	2.23 (-1.03, 5.61)	0.134	0.536
**Sizes**						
<100μm	6.42 (-2.69, 16.4)	0.762	0.762	4.3 (-2.69, 11.8)	0.170	0.255
100-200μm	2.75 (-1.32, 6.98)	0.395	0.761	6.51 (0.43, 13.0)	**0.046**	0.138
≥200μm	0.530 (-4.61, 5.95)	0.507	0.760	5.78 (-3.75, 16.3)	0.596	0.596
**Main polymer types**						
PA	7.04 (0.23, 14.3)	**0.048**	0.096	7.15 (-4.31, 20.0)	0.836	0.836
PET	2.27 (-2.12, 6.84)	0.375	0.429	2.45 (-4.07, 9.42)	0.478	0.637
PE	5.64 (1.33, 10.1)	**0.029**	0.096	7.61 (1.21, 14.4)	**0.037**	0.148
PP	4.63 (-1.26, 10.8)	0.427	0.429	3.26 (-1.26, 7.99)	0.335	0.637

Abbreviations: MDA, malondialdehyde; 8-OHdG, 8-hydroxy-2′-deoxyguanosine; PA, polyamide; PET, polyester; PE, polyethylene, PP, polypropylene. Variables such as magnitudes of fecal shapes were coded as dummy variables (i.e., 1 for bars microplastics and 0 for others shapes microplastics) and linear mixed-effect models were conducted, with adjustment for age (continuous), BMI (continuous), sleep duration (continuous), and physical activity (yes/no) as fixed effect covariates.

Table S4. Associations of the shape, size, and chemical types of fecal MPs with lung function

Variables	β (95%CI) in FVC	*P*	FDR	β (95%CI) in FEV_1	*P*	FDR	β (95%CI) in PEF	*P*	*FDR*
**Shapes**									
sheet	-0.09(-0.234, 0.054)	0.207	0.207	-0.008(-0.215, 0.199)	0.785	0.785	-0.069(-0.156, 0.018)	0.340	0.680
fragment	-0.043(-0.124, 0.038)	0.103	0.207	-0.010(-0.136, 0.116)	0.432	0.785	-0.026(-0.108, 0.056)	0.297	0.680
pellet	-0.029(-0.114, 0.056)	0.116	0.207	-0.004(-0.146, 0.138)	0.521	0.785	-0.097(-0.841, 0.647)	0.809	0.703
fiber	-0.089(-0.215, 0.037)	0.163	0.207	-0.072(-0.274, 0.130)	0.741	0.785	-0.038(-0.149, 0.074)	0.527	0.809
**Sizes**									
<100μm	-0.060(-0.145, 0.025)	0.173	0.259	-0.035(-0.166, 0.096)	0.626	0.66	-0.032(-0.744, 0.680)	0.938	0.938
100-200μm	-0.036(-0.114, 0.042)	0.142	0.259	-0.034(-0.159, 0.091)	0.581	0.66	-0.054(-0.124, 0.015)	0.132	0.396
≥200μm	-0.013(-0.128, 0.102)	0.297	0.297	-0.030(-0.162, 0.102)	0.666	0.66	-0.032(-0.758, 0.694)	0.936	0.938
**Main polymer types**									
PA	-0.0145(-0.042, 0.013)	0.076	0.304	-0.002(-0.259, 0.255)	0.788	0.788	-0.022(-0.237, 0.194)	0.772	0.918
PET	-0.056(-0.142, 0.030)	0.219	0.352	-0.056(-0.187, 0.075)	0.430	0.781	-0.043(-0.932, 0.846)	0.918	0.918
PE	-0.081 (-0.214, 0.052)	0.352	0.352	-0.058 (-0.276, 0.160)	0.586	0.781	-0.014 (-0.035, 0.007)	0.105	0.420
PP	-0.013 (-0.120, 0.094)	0.331	0.352	-0.003 (-0.142, 0.136)	0.374	0.781	-0.015 (-0.183, 0.153)	0.402	0.804

Abbreviations: FVC, forced vital capacity; FEV_1, forced expiratory volume in 1 s; PEF, peak expiratory flow; PA, polyamide; PET, polyester; PE, polyethylene, PP, polypropylene. Variables such as magnitudes of fecal shapes were coded as dummy variables (i.e., 1 for bars microplastics and 0 for others shapes microplastics) and linear mixed-effect models were conducted, with adjustment for age (continuous), BMI (continuous), sleep duration (continuous), and physical activity (yes/no) as fixed effect covariates.

Table S5. Associations of the shape, size and chemical types of fecal MPs with blood pressure

Variables	β (95%CI) in SBP	*P*	*FDR*	β (95%CI) in SBP	*P*	*FDR*	β (95%CI) in SBP	*P*	*FDR*	β (95%CI) in SBP	*P*	*FDR*
**Shapes**												
sheet	-0.466(-1.75, 0.818)	0.327	0.485	-0.174(-0.675, 0.327)	0.545	0.726	-1.11(-6.59, 4.37)	0.712	0.712	-0.341(-1.51, 0.823)	0.442	0.823
fragment	-0.110(-4.49, 4.27)	0.956	0.956	-0.128(-2.14, 1.88)	0.442	0.726	-0.113(-3.05, 2.82)	0.499	0.712	-0.765(-4.02, 2.49)	0.663	0.823
pellet	-0.925(-2.22, 0.374)	0.364	0.485	-0.272(-0.998, 0.454)	0.103	0.412	-0.435(-3.2, 2.33)	0.430	0.712	-0.219(-0.829, 0.391)	0.220	0.823
fiber	-0.798(-1.97, 0.377)	0.314	0.485	-0.658(-5.57, 4.25)	0.802	0.802	-0.174(-4.23, 3.88)	0.661	0.712	-0.377(-6.80, 6.04)	cc	0.823
**Sizes**												
<100μm	-0.387(-1.71, 0.931)	0.396	0.480	-0.211(-3.45, 3.03)	0.902	0.902	-0.211(-0.952, 0.530)	0.223	0.669	-0.941(-4.34, 2.45)	0.599	0.599
100-200μm	-0.158(-1.31, 0.996)	0.321	0.480	-0.127(-0.447, 0.193)	0.129	0.387	-0.425(-3.71, 2.86)	0.734	0.738	-0.387(-3.37, 2.60)	0.414	0.599
≥200μm	-0.367(-2.66, 1.93)	0.480	0.480	-0.998(-3.99, 2.00)	0.864	0.902	-0.279(-3.57, 3.01)	0.738	0.738	-1.09(-4.24, 2.06)	0.550	0.599
**Main polymer types**												
PA	-0.255(-1.78, 1.27)	0.579	0.726	-0.196(-0.91, 0.52)	0.542	0.837	-0.413(-1.37, 0.547)	0.301	0.754	-0.630(-7.80, 6.54)	0.935	0.935
PET	-0.217(-1.80, 1.37)	0.406	0.726	-0.213(-0.54, 0.110)	0.244	0.837	-0.496(-1.82, 0.825)	0.377	0.754	-0.706(-4.20, 2.79)	0.714	0.935
PE	-0.517 (-3.26, 2.23)	0.631	0.726	-0.604 (-5.21, 4.00)	0.912	0.837	-0.127 (-4.01, 3.76)	0.847	0.847	-0.745 (-3.36, 1.87)	0.609	0.935
PP	-0.771 (-3.86, 2.32)	0.726	0.726	-0.477 (-3.19, 2.24)	0.628	0.837	-0.365 (-3.72, 2.99)	0.764	0.847	-0.405 (-4.98, 4.17)	0.895	0.935

Abbreviations: SBP, systolic blood pressure; DBP, diastolic blood pressure; PP, pulse pressure; MAP, mean arterial pressure; PA, polyamide; PET,

polyester; PE, polyethylene, PP, polypropylene. Variables such as magnitudes of fecal shapes were coded as dummy variables (i.e., 1 for bars microplastics and 0 for others shapes microplastics) and linear mixed-effect models were conducted, with adjustment for age (continuous), BMI (continuous), sleep duration (continuous), and physical activity (yes/no) as fixed effect covariates.

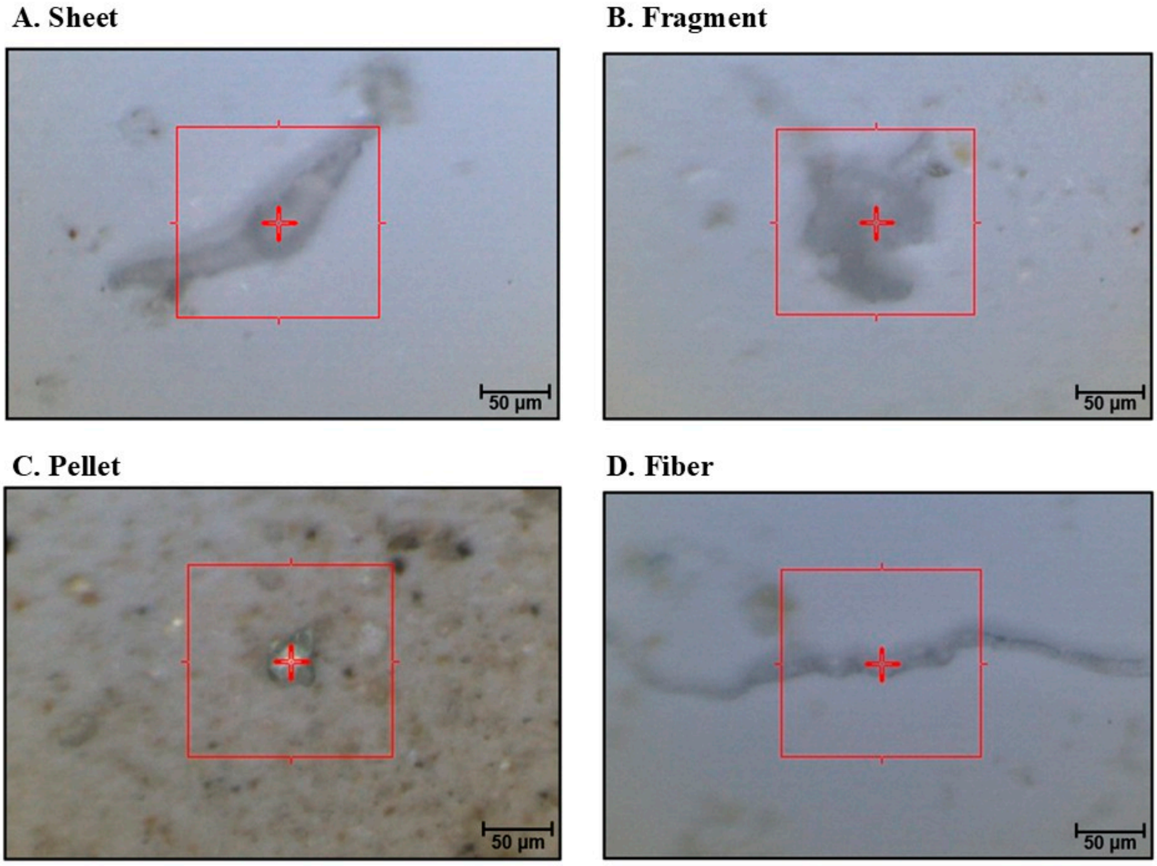

Figure S1. Four shapes of microplastics detected in feces.

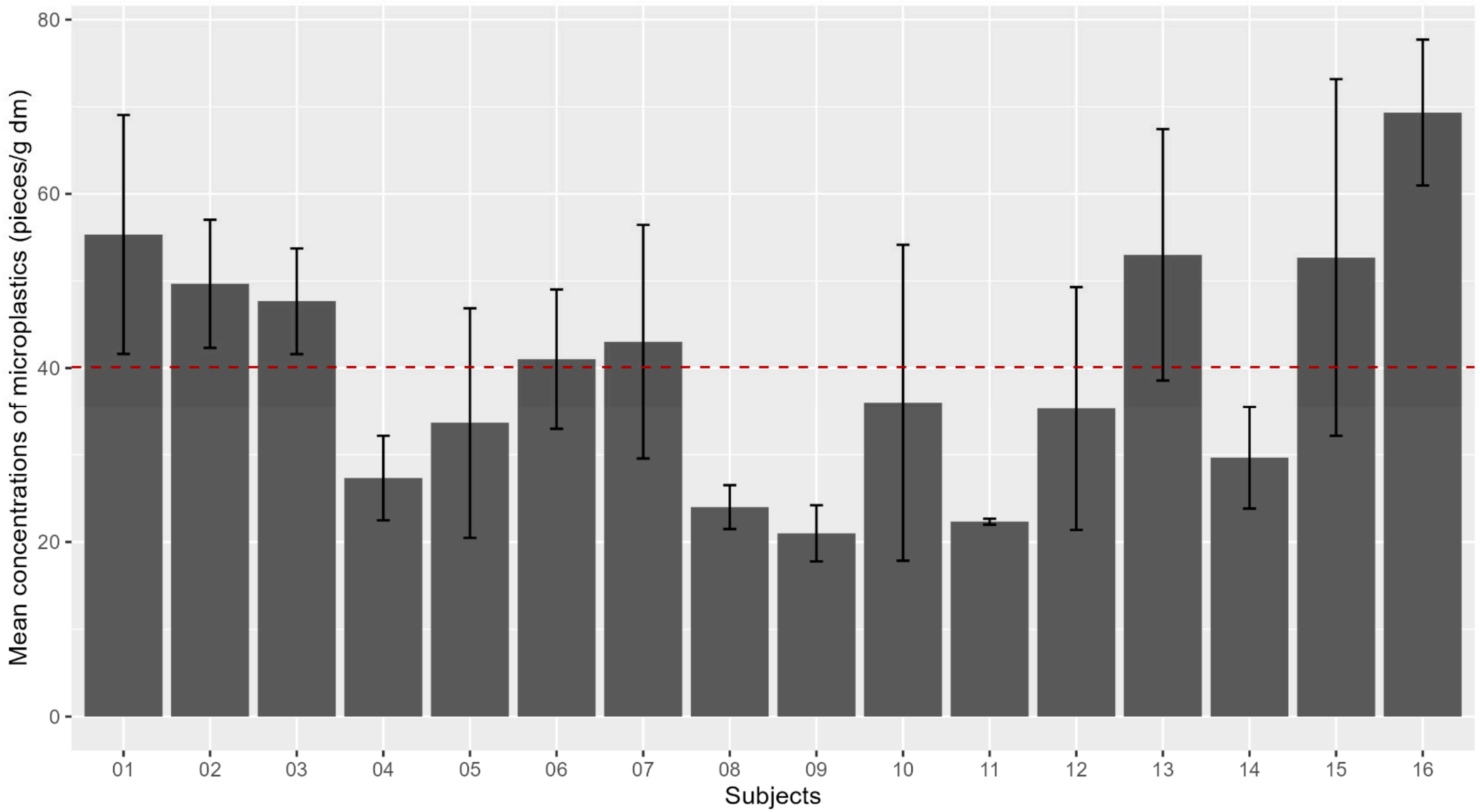

Figure S2. Average concentrations of microplastics in feces by16 young male college students with three visits (N=48). The line chart was plotted to characterize distributions of fecal microplastics levels between persons. Bars represented mean level for each subject labeled 01 to 16. The red dotted line was the overall mean concentration of 48 samples with a value being 40.1 pieces/g dm.

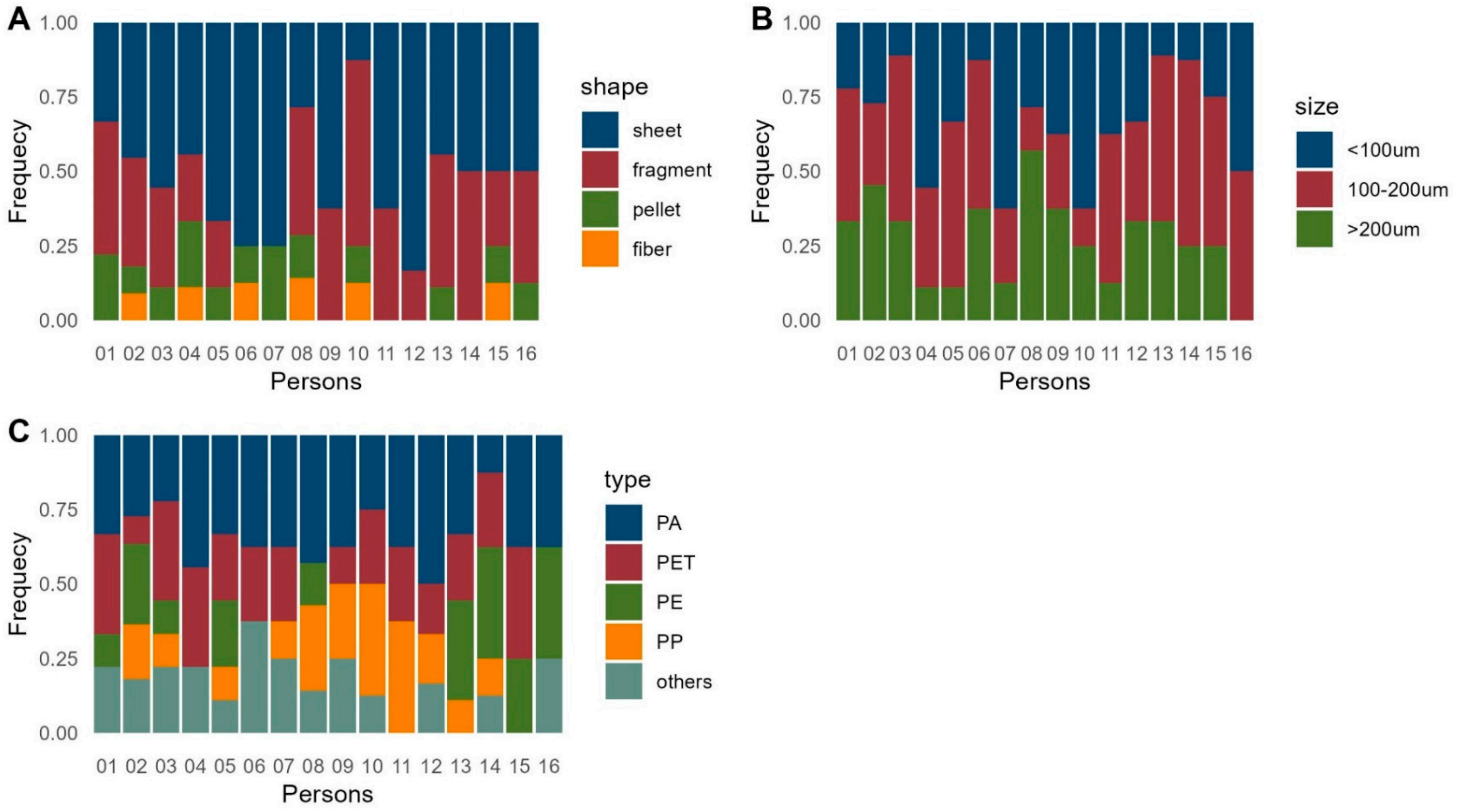

Figure S3. Distributions for the shape, size, and chemical type of fecal microplastics in 16 male college students with three-round visits. Percentage bar graph depicted between-person frequency distributions for the shape, size, and chemical types of fecal microplastics. PA, polyamide; PET, polyester; PE, polyethylene, PP, polypropylene. Those with relative proportions less than 10% were classified as others, including polyisobutylene (PIB), polylactic acid (PLA), cellulose nitrate (CN), ethylene vinyl acetate copolymer (EAA).
